# Phytochemical and In-Vivo Anti-Arthritic Significance of *Aloe thraskii* Baker in Combined Therapy with Methotrexate in Adjuvant-Induced Arthritis in Rats

**DOI:** 10.3390/molecules26123660

**Published:** 2021-06-15

**Authors:** Rania M. Kamal, Manal M. Sabry, Zeinab Y. Aly, Mohamed S. Hifnawy

**Affiliations:** 1 Department of Pharmacognosy, Faculty of Pharmacy, Cairo University, Cairo 11562, Egypt; raniakamal93@gmail.com (R.M.K.); mohamed.hefnawy@pharma.cu.edu.eg (M.S.H.); 2 Department of Biochemistry, National Organization for Drug Control and Research (NODCAR), Giza 35521, Egypt; zeinabyouaef65@gmail.com

**Keywords:** *Aloe thraskii*, phytochemical, anti-arthritic, combined methotrexate therapy, complete Freund’s adjuvant

## Abstract

Unlike other widely known *Aloe* species used for treatment of rheumatoid arthritis, this species suffers from a lack of sufficient studies on its biological and chemical characters. This is what drove us to perform this work to evaluate the in vivo anti-arthritic potential of its leaf ethanolic extract. The in vivo anti-arthritic activity of the leaf ethanolic extract at 100 and 200 mg/kg/day b.wt. was evaluated alone and in combination with methotrexate (MTX) using complete Freund’s adjuvant. Serum levels of rheumatoid factor, anti-cyclic citrullinated peptide (anti-CCP), cytokines pro-inflammatory marker, inflammatory mediator serum levels, and oxidative stress mediators were analyzed, in addition to liver function. Orientin, isoorientin, β-sitosterol, its palmitate and its glucoside were isolated. The combined therapy of MTX and the leaf ethanolic extract (especially at 200 mg/kg b.wt.) group showed better activity compared to MTX alone. Moreover, the combined therapy provided additional benefits in lowering the liver toxicity by comparison to MTX alone. We concluded that a synergetic combination of the leaf ethanolic extract and MTX is beneficial in the management of rheumatoid arthritis with fewer side effects on liver function, as well as the possibility of the leaf extract to stand alone as an effective natural anti-arthritic agent.

## 1. Introduction

Rheumatoid arthritis (RA), a chronic joints inflammation, is described as an autoimmune ailment affecting 1–2% of human beings all over the world, especially females [1]. The resulting joint damage, musculoskeletal deficits and pain provokes physical disabilities as well as physiological distress, leading to the subsequent social isolation of the patient [2].

RA is accompanied by the development of different antigens which, in turn, activate certain inflammatory mediators (such as tumor necrosis factor-alpha (TNF-α) and interleukins (IL-1, IL-6, IL-10) [3,4]). Likewise, the activation of the pro-inflammatory cytokine nuclear transcription factor kappa B (NF-ĸB) contributes to the development of chronic inflammation in RA [5], while IL-10 participates in inhibiting inflammatory and autoimmune processes [3]. Rheumatoid factor (RF) and anticyclic citrullinated peptide (anti-CCP) antibodies are the most predictive markers for RA diseases [6].

Various medications are used in an attempt to keep the normal articulations functioning and retard or even stop the progressive inflammatory processes or reduce the pain. Methotrexate (MTX) is a known antirheumatic drug used for moderate to severe RA; however, hepatotoxicity is the main constraint to its use [7,8,9].

On the other hand, such disadvantages could be bypassed by using dietary and herbal preparations with anti-inflammatory and antiarthritic characteristics, offering a new opportunity. Various studies [10,11] have been conducted using the outcome of both MTX and natural products in combined therapy, to attain a more efficient drug with reduced side effects.

Genus *Aloe* is a large genus comprised of more than 500 species [12]. It belongs to the family Asphodelaceae, a monocotyledon family, comprised of about 40 genera and 900 species of perennial temperate and subtropical succulent herbs [13,14].

In this context, *Aloe* species exhibited different pharmacological activities, including anti-inflammatory [15,16,17], antiarthritic, wound healing, hepatoprotective [16,17,18], cytotoxic [17], antidiabetic [19], immunostimulant and antimicrobial activities [16,17,20]. Several phytochemical constituents were investigated viz; anthraquinones, naphthoquinones, chromones, coumarins, pyrans and pyrons, flavonoids and phenolic acids, sterols and triterpenes, alkaloids and polysaccharides [17,18].

*Aloe thraskii* Baker (commonly known as Dune *Aloe*, Coast *Aloe* or Strand *Aloe*) grows in sand dunes and along the east coast of South Africa [20]. In South Africa, the fresh leaves and sap were traditionally used to treat snake bites, wounds, and sores [21]. From our best review of the current literature, few reports about the phytochemical and biological studies of *Aloe thraskii* Baker were found. Wyk et al., (1995) [22] stated that chrysophanol, aloesaponarin I, aloesaponarin II, asphodelin, aloesaponol I and aloesaponol II were detected in the roots via HPLC. Concerning the biological activities, the leaf exudate possessed a moderate effect on lipid peroxidation but no antioxidant activity [23], a stimulant effect on fertility [24] and an antiemetic effect [25].

In the present study, a phytochemical analysis, as well as the influence of *Aloe thraskii* Baker ethanolic leaf extract on the reduction of rheumatoid arthritis when used in combined therapy with MTX, was evaluated. Different *Aloe* species were reputed for their antiarthritic and hepatoprotective activities. Therefore, the ability of the plant under study to evade the hepatocellular toxicity produced by MTX therapy is studied using complete Freund’s adjuvant-induced arthritis rat model.

## 2. Results

### 2.1. Characterization of Isolated Compounds

Structure elucidation of isolated compounds was based on UV and 1D-2D NMR spectroscopy, in addition to comparison to the reported data.

**Data of compound 1:** white amorphous powder, R_f_ = 0.375 (*n*-hexane/ethyl acetate 9.5:0.5). ^1^H-NMR (400 MHz, CDCl_3_) δ: 0.61 (3H, *s*, Me-18), 0.95 (3H, *s*, Me-19), 0.72 (3H, *d*, *J* = 7 Hz, Me-26), 0.75 (3H, *d*, *J* = 6.3 Hz, Me-27), 0.78 (3H, *d*, *J* = 5.7 Hz, Me-21), 0.87 (3H, *t*, *J* = 6.3, 7.5 Hz, Me-29), 5.31 (1H, *brs*, H-6), 4.57 (1H, *m*, H-3). ^13^C-NMR (100 MHz, CDCl_3_) 11.6 (C-18), 19.7 (C-19), 18.6 (C-21), 18.91 (C-26), 19.05 (C-27) and 11.75 C-29 methyl carbons; 29.2 (C-2), 39.5 (C-7), 20.6 (C-11), 23.8 (C-15), 25.5 (C-23), 28.7 (C-25) and 22.6 (C-28), 139.4 (C-5), 122.1 (C-6), 14.4 (CH_3_ ter.), 173.4 (C-1′), 31–36 correspond to (CH_2_)_n_ of sterol and long chain of fatty acid.

**Data of compound 2:** white needle crystals, R_f_ = 0.46 (*n*-hexane/ethyl acetate solvent 9:1).^1^H-NMR (400 MHz, CDCl_3_) 0.61 (3H, *s*, Me-18), 0.95 (3H, *s*, Me-19), 0.72 (3H, *d*, *J* = 7, Me-26), 0.75 (3H, *d*, *J* = 6.3, Me-27), 0.78 (3H, *d*, *J* = 5.7, Me-21), 0.87 (3H, *t*, *J* = 6.3,7.5, Me-29), 5.37 (1H, *brs*, H-6) and 3.55 (1H, *m*, H-3).

**Data of compound 3:** white crystals, R_f_ = 0.3 (CH_2_Cl_2_/CH_3_OH 9:1). ^1^H-NMR (400 MHz, DMSO) 0.65 (3H, *s*, Me-18), 0.95 (3H, *s*,Me-19), 0.79 (3H, *d*, *J* = 7 Hz, Me-26), 0.83 (3H, *d*, *J* = 6.3 Hz, Me-27), 0.90 (3H, *d*, *J* = 5.7 Hz, Me-21), 1.16 (3H, *t*, *J* = 6.3, 7.5 Hz, Me-29), 5.34 (1H, *brs*, H-6), 3.65 (1H, *m*, H-3), 4.22 (1H, *d*, *J* = 7.7 Hz, H-1′). ^13^C-NMR (100 MHz, DMSO) 11.6 (C-18), 19.7 (C-19), 18.6 (C-21), 18.91 (C-26), 19.05 (C-27), 11.75 (C-29), 29.2 (C-2), 39.5 (C-7), 20.6 (C-11), 23.8 (C-15), 25.5 (C-23), 28.7 (C-25), 22.6 (C-28), 31.4 (C-8), 36.2 (C-10), 36.8 (C-12), 35.5 (C-20), 33.3 (C-22), 41.8 (C-4), 49.6 (C-9), 45.1 (C-13), 56.2 (C-14) and 55.4 (C-17), 141.1 (C-5), 121.9 (C-6), Sugar: 101.2 (C-1′), 70.1 (C-2′), 74.4 (C-3′), 77.0 (C-4′), 77.4 (C-5′) and 61.4 (C-6′).

**Data of compound 4:** yellow powder, R_f_ = 0.30 (ethyl acetate/CH_3_OH/H_2_O/formic acid 100:16.5:13.5:0.2). UV (CH_3_OH): 348, 270 and 255. ^1^H-NMR (400 MHz, DMSO) 6.48 (1H, *s*, H-8), 6.67 (1H, *s*, H-3), 6.89 (1H, *d*, *J* = 8 Hz, H-5′), 7.41 (1H, d, *J* = 7.8 Hz, H-6′), 7.43 (1H, *d*, *J* = 7.8 Hz, H-2′), 4.66 (1H, *d*, *J* = 8 Hz, H-1′’), 3–4.1 (sugar protons). ^13^C-NMR (100 MHz, DMSO) Aglycone: .162 (C-2), 103.1 (C-3), 182.2 (C-4), 161.8 (C-5), 108.5 (C-6), 164.0 (C-7), 94.0 (C-8), 156.8 (C-9), 103.7 (C-10), 121.7 (C-1′), 113.6 (C-2′), 146.2 (C-3′), 150.3 (C-4′), 116.3 (C-5′), 119.5 (C-6′). Sugar: 71.7 (C-1′’), 74.6 (C-2′’), 78.8 (C-3′’), 76.6 (C-4′’), 82.1 (C-5′’), 61.9 (C-6′’). The HMBC of compound **4** showed a correlation between anomeric proton at δ 4.66 and carbons at (78.8, 108.5 and 164.0) for C-3′’, C-6 (downfielded from the normal value 99) and C-7, respectively), which confirmed the position of glycosylation at C-6. The HSQC of compound **4** exhibited a correlation between anomeric proton at (δ 4.66) and a carbon at (δ 71.80) which assigned C-1′’, which confirmed a C-glycosylation.

**Data of compound 5:** yellow powder, R_f_ = 0.15 (ethyl acetate/CH_3_OH/H_2_O/formic acid 100:16.5:13.5:0.2). UV (CH_3_OH): 348, 270 and 255; ^1^H-NMR (400 MHz, DMSO) 6.51 (1H, *s*, H-6), 6.65 (1H, *s*, H-3), 6.88 (1H, *d*, *J* = 8 hz, H-5′), 7.40 (1H, d, *J* = 7.8 Hz, H-6′), 7.41 (1H, *d*, *J* = 7.8 Hz, H-2′), 4.60 (1H, *d*, *J* = 8 Hz, H-1′’), 3–4.1 (sugar protons).^13^C-NMR (100 MHz, DMSO): Aglycone δ: 156.8 (C-2), 182.5 (C-4), 161.1 (C-5), 102.9 (C-6), 164.0 (C-7), 109.4 (C-8), 161.1 (C-9), 103.6 (C-10), 121.9 (C-1′), 119.5 (C-2′), 146.4 (C-3′), 150.4 (C-4′), 116.6 (C-5′), 113.5 (C-6′). Sugar: 73.3 (C-1′’), 72.4 (C-2′’), 70.7 (C-3′’), 79.3 (C-4′’), 82.1 (C-5′’), 62.0 (C-6′’).

The HMBC of compound **5** demonstrated a correlation between anomeric proton (at δ: 4.60) and carbon atoms at (δ: 70.7, 82.1 and 109.4) assigned for C-3′’, C-5′’ and C-8 (down fielded from normal value 93), respectively, which confirmed the glycosylation at C-8. Likewise, HSQC showed a correlation between an anomeric proton (at δ: 4.60) and C-1′’ (at δ: 73.30), which confirmed a C-glycosylation.

### 2.2. Identification of Compounds

**Figure 1:**
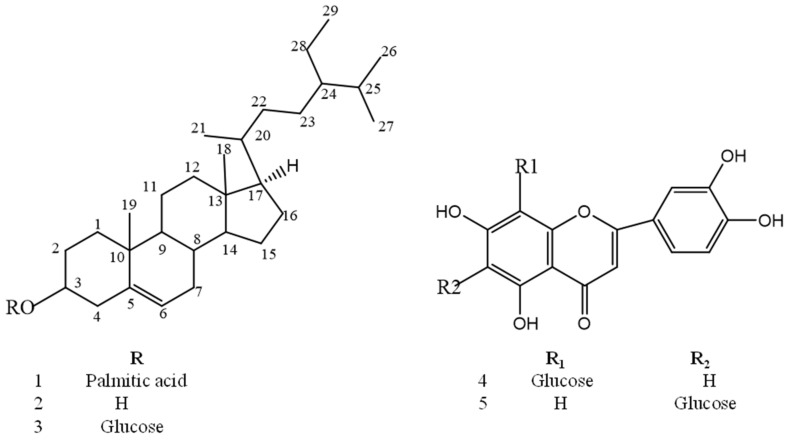
Structures of compounds separated from *Aloe thraskii* Baker leaves.

From the leaves of *Aloe thraskii* Baker, β-Sitosterol palmitate (**1**) [26], β-sitosterol (**2**) [27], β-sitosterol-3-*O*-β-d-glucoside (**3**) [28], luteolin-6-C-β-glucopyranoside (known as isoorientin (**4**) [18]) and luteolin-8-C-β-glucopyranoside (known as orientin (**5**) [18]) were isolated for the first time. Their structures are displayed in Figure 1.

### 2.3. Biological Study

#### 2.3.1. Evaluation of Median Lethal Dose (LD_50_)

No mortality was recorded after careful observation of animals for 14 days. Accordingly, a maximum safety dose 200 mg/kg (1/10th of 2000 mg/kg) was chosen with two folds interval descending dose levels: 100 and 200 mg/kg body weight of the test rat (OECD, 2001).

#### 2.3.2. Effect of Different Treatments on the Levels of Diagnostic Markers of Rheumatoid Arthritis (RF and Anti-CCP)

Rheumatoid factor (RF) and the more specific anticyclic citrullinated peptide (anti-CCP) antibodies are regarded as the most distinct markers for RA diagnosis.

From Table 1, The RF serum level in untreated arthritic rats (negative control group) increased by 263.45%, while anti-CCP showed a 456.54% increase compared to normal control group.

Compared to negative control group, the level of RF was significantly decreased by 14.4% in the case of 200 mg/Kg b.wt. of the ethanolic extract, decreased by 40.3% in the MTX treated arthritic group, while a more substantial reduction of 47% and 52% was observed in case of MTX combined therapy with the two dose levels of ethanolic extract, respectively.

Likewise, compared to negative control group, the level of anti-CCP was significantly decreased by 31.4% in the case of 200 mg/Kg b.wt. of the ethanolic extract, decreased by 47.6% in MTX treated arthritic group, while a more substantial reduction of 52.6% and 64.6% was observed in case of MTX combined therapy with the two dose levels of ethanolic extract, respectively.

#### 2.3.3. Effect of Different Treatments on the Levels of Markers of Inflammation (NF-ĸB, TNF-α and IL-10)

From the results in Table 2, it was deduced that the proinflammatory markers NF-ĸB and TNF-α serum levels elevated to 364.38% and 370%, respectively, in negative control rats against the normal control.

Compared to the negative control group, the level of NF-ĸB and TNF-α was significantly reduced by 59.2% and 46.1%, respectively, in MTX treated arthritic group, by 35% and 47.1%, respectively, in the case of 200 mg/kg b.wt. of the ethanolic extract, while a significant reduction of 63.69% and 59.9%, respectively, was observed in the case of MTX combined therapy with 200 mg/kg b.wt. of the ethanolic extract.

Concerning the inflammatory mediator IL-10, its serum level in arthritic negative control decreased by 44.69% against the normal control rats. Compared to the negative control group, its serum level significantly was elevated by 64.2% in the MTX treated arthritic group and by 75.7% in the case of 200 mg/Kg b.wt. of the ethanolic extract, while a significant increase of 82% was observed in case of MTX combined therapy with 200 mg/kg b.wt.

#### 2.3.4. Effect on Oxidative Stress Mediators

From the results recorded in Table 3, it was deduced that the serum level of oxidative stress mediators TAC and GSH in negative control rats reduced by 17.3% and 21.4%, respectively, while that of MDA was elevated by 22.7% against the normal control rats.

In the MTX treated group, the serum levels of TAC and GSH were significantly reduced by 33.8% and 39.9%, respectively, against the negative control group. However, the MDA serum level was elevated by 33.7%.

On the other hand, the animal group receiving 200 mg/kg b. wt of the ethanolic extract of *A. thraskii* leaves showed a remarkable increase of 15% and 21% of TAC and GSH serum levels, respectively, and a significant reduction of 39.5% of MDA serum level.

The combined therapy of MTX and 200 mg/kg b.wt. of the leaf ethanolic extract of *A. thraskii* exhibited a significant decrease of 11.8% and 18% of TAC and GSH serum levels, respectively, and a significant increase of 15.5% of MDA serum level.

#### 2.3.5. Markers of Liver Function (AST, ALT and ALP):

In AFC-induced arthritic rats, serum levels of AST, ALT and ALP resulted in a significant elevation to 122%, 137.5% and 64.8%, respectively, at *p* < 0.05 against the normal control rats (Table 4). A remarkable significant elevation in the liver enzymes by 31.1, 37.8 and 62.4%, respectively, was observed in MTX treated rats at *p* < 0.05 against negative control.

On the other hand, the three liver enzymes showed a remarkable significant reduction of 40.7%, 36.1% and 32.8%, respectively, in the leaf ethanolic extract 200 mg/kg b.wt. treated group. The combined therapy of MTX and the leaf ethanolic extract at the two doses has provided additional benefits in lowering the three enzyme levels, in comparison to MTX-treated rats.

#### 2.3.6. Total Bilirubin and Total Protein

From the results in Table 5, it was deduced that the total bilirubin serum level was elevated by 80.7% in the negative control against the normal control rats and by 58.9% in the MTX treated arthritic group against the negative control group. However, rat groups receiving 100 and 200 mg/Kg of the leaf ethanolic extract illustrated a better effect represented by a significant reduction by 19% and 31%, respectively. In the case of MTX combined therapy with the two dose levels of the leaf ethanolic extract, a significant increase of 43.1% and 27.1%, respectively, was observed.

Regarding the results of total protein serum level, it was decreased by 22.7% in negative control rats when compared to the normal control and by 18% in the MTX treated arthritic group when compared to the negative control group. However, rat groups receiving 100 and 200 mg/kg b.wt. of the leaf ethanolic extract showed a better effect represented in a significant elevation of 6.2% and 22%, respectively. In the case of MTX combined therapy with 100 and 200 mg/kg b.wt. of the leaf ethanolic extract, a significant decrease of 11.3% and 4.2%, respectively, was observed.

## 3. Discussion

The current phytochemical investigation resulted in the separation and the structure elucidation of five compounds from the leaf ethanolic extract of *A. thraskii* for the first time. The isolated compounds are β-Sitosterol palmitate (**1**), β-sitosterol (**2**), β-sitosterol-3-*O*-β-d-glucoside (**3**), luteolin-6-C-β-glucopyranoside (known as isoorientin (**4**)) and luteolin-8-C-β-glucopyranoside (known as orientin (**5**)).

The toxicity study showed no mortality. As a result, the leaf ethanolic extract is considered safe. Accordingly, the maximum safety dose of 200 mg/kg was selected with descending two-fold interval dose levels (i.e., 100 mg/kg and 200 mg/kg body weight of the tested animals [29].

The ability of the leaf ethanolic extract to exhibit an antiarthritic activity, as well as to evade the hepatocellular toxicity produced by methotrexate therapy, is being studied using the CFA-induced arthritis rat model.

The CFA-induced arthritis rat model is considered the most appropriate for the simulation of rheumatoid arthritis. RA was associated with complicated reactions of various inflammatory, chemotaxis and phagocytosis processes, resulting in cartilage and bone degradation [30]. In CFA-induced arthritis, the negative control rats showed increased serum levels of the diagnostic RA markers: RF and anti-CCP. Treatment with the leaf ethanolic extract at the two-dose levels suppressed the serum levels of RF and anti-CCP in a dose dependent way. This antiarthritic potential was confirmed by significantly decreased production of the proinflammatory markers NF-ĸB and TNF-α serum levels and a significantly increased level of an inflammatory mediator (IL-10). Alongside this, a remarkable elevation of the oxidative stress mediators TAC and GSH (in addition to a decrease of a lipid peroxidation marker MDA level) were observed, giving an indication of the anti-inflammatory and antioxidant mechanism of the plant. Another promising biological effect of the ethanolic extract was the hepatoprotective activity, as proved by the liver function enzymes AST, ALT and ALP, in addition to total bilirubin and total protein.

MTX is considered as the drug of choice in the case of RA, although the developed adverse effects (especially its hepatotoxicity) greatly limit its use [11]. A combination therapy of MTX with a plant extract can offer a ray of hope in therapy, as it could accomplish the best action with minimal side effects.

The combined therapy of MTX and the leaf ethanolic extract (especially at 200 mg/Kg b.wt.) groups showed better antirheumatic activity as compared to MTX alone. This result was emphasized through an increased inhibition in the serum levels of RF and anti-CCP in the pro-inflammatory markers NF-ĸB and TNF-α, as well as a more elevated percent of serum MDA. Moreover, the combined therapy has provided additional benefits in lowering the liver toxicity by comparison to MTX alone.

Finally, the current investigation suggested that the leaf ethanolic extract of *A. thraskii* possesses an antiarthritic effect, reported for the first time. This action could be due to the isolated compounds which have reported anti-inflammatory and antiarthritic activities [31,32,33,34]. β-sitosterol and β-sitosterol-3-*O*-β-d-glucoside moderate macrophage polarization and attenuate rheumatoid arthritis in mice [34]. Orientin inhibits certain inflammatory factors such as prostaglandin PGE2, tumor necrosis factor-alpha TNF-α and interleukin IL-1β serum levels and inflammatory mediators in mast cells [32,33]. Isoorientin is considered to be a selective cyclooxygenase-2 (COX-2) inhibitor [31]. Moreover, the isolated compounds could further explore the possible antirheumatic action. This activity was mediated through antioxidant, anti-inflammatory and hepatoprotective mechanisms, which could delay or inhibit the development of RA processes.

## 4. Materials and Methods

### 4.1. Plant Material

This work was done on fresh *Aloe thraskii* Baker leaves. The plant was collected from August to April 2016 from El-Hosary Botanical Garden, Giza, Egypt (latitude 29°58′22.5”N and longitude 30°56′38.1”E) and cultivated in the Experimental Plant Station for Aromatic and Medicinal plants, Faculty of Pharmacy, Cairo University, Giza, Egypt (latitude 30°1′49.9074”N, and longitude 31°11′43.8894”E). Botanical identification was kindly performed by Dr. Mohamed Gebaly, senior botanist and taxonomist and confirmed by Eng. Therese Labib, consultant in Orman Garden and National Gene Bank, Ministry of Agriculture. An herbarium bord (code No.1–6-2018) was placed at the Museum of Pharmacognosy Department, Faculty of Pharmacy, Cairo University.

### 4.2. Phytochemical Analysis

#### 4.2.1. Chemicals and Chromatographic Techniques

Solvents of analytical grade viz., ethanol, methanol, *n*-hexane, methylene chloride, ethyl acetate, *n*-butanol and distilled water were used for the extraction, fractionation and chromatographic elution processes. Various chromatographic columns were utilized: Silica gel 60 (Fluka, 70–230 mesh and 35–70 mesh, ASTM; Taufkirchen, Germany), Sephadex LH-20 (Pharmacia Fine Chemicals AB, Uppsala, Sweden) and Diaion (E-Merck Darmstadt, Germany). Thin layer chromatography (TLC) was performed using precoated silica 60 F_254_ plates (Macherey Nagel, Germany. ^1^H-NMR and ^13^C-NMR spectroscopy were conducted on a Bruker 400 MHz nuclear magnetic resonance spectrometer utilizing CDCl_3_ and DMSO for sample preparation.

#### 4.2.2. Extraction and Fractionation Procedures

Eight kg of fresh leaves were cut into small pieces and cold macerated in 95% ethanol till exhaustion, and the obtained ethanolic extract was dried under reduced pressure yielding 160 g. Forty g was kept for biological studies and the rest was suspended in distilled water (250 mL) and successively partitioned with petroleum ether (6 × 400 mL), methylene chloride (5 × 400 mL), ethyl acetate (7 × 500 mL) and *n*-butanol saturated with water (8 × 400 mL). Each fraction was dried under reduced pressure, yielding 8, 2, 7 and 44 g of each fraction, respectively.

#### 4.2.3. Isolation of Compounds

The petroleum ether fraction (6 g) was added to a silica gel 60-column chromatography (3D × 30L cm, 150 g). A gradient elution was performed using petroleum ether/methylene chloride and methylene chloride/ethyl acetate mixtures. Four main fractions were collected.

One fraction (30% CH_2_Cl_2/_petroleum ether) was subjected to fractionation on silica gel 60 column using petroleum ether/ethyl acetate gradient elution, which resulted in the isolation of compound (**1**) (15 mg, white amorphous powder, R_f_ = 0.375 in *n*-hexane-ethyl acetate 9.5:0.5). A second fraction (70–80% CH_2_Cl_2_/ethyl acetate) was subjected to fractionation on silica gel 60 column using petroleum ether/ethyl acetate gradient elution, which resulted in the isolation of compound (**2**) (10 mg, white needle crystals, R_f_ = 0.46 in *n*-hexane-ethyl acetate solvent 9:1).

The methylene chloride fraction (1 g) was chromatographed on a silica column chromatography (2.5D × 15L cm, silica gel 60). A gradient elution was performed using CH_2_Cl_2_, CH_2_Cl_2_/CH_3_OH mixtures. Two main fractions were obtained. One fraction (50% to 10% CH_2_Cl_2_-CH_3_OH) was subjected to fractionation on silica gel 60 column using CH_2_Cl_2_-ethyl acetate gradually as an eluent, which resulted in the isolation of one pure compound, namely (**3**) (30 mg, white crystals, R_f_ = 0.3 in CH_2_Cl_2_-CH_3_OH 9:1).

About a half weight of the *n*-butanol fraction (20 g) was chromatographed on a column chromatography (3.5D × 25L cm, Diaion). A decreasing gradient elution was performed using distilled H_2_O and H_2_O/CH_3_OH mixtures. Five fractions were obtained. One fraction (50% H_2_O/CH_3_OH) was subjected to repeated column chromatography on Sephadex LH-20 (2.5 × 15 cm) using H_2_O-CH_3_OH (1:1), resulting in the isolation of compounds (**4**) (7 mg, yellow powder, R_f_ 0.30 in S_4_) and (**5**) (5 mg, yellow powder, R_f_ = 0.15 in ethyl acetate–methanol–water–formic acid 100:16.5:13.5:0.2).

### 4.3. Biological Study

#### 4.3.1. Experimental Animals

Adult male albino Wistar rats of 130–150 g body weight (aged 8 weeks) were used to determine the LD_50_, antiarthritic, anti-inflammatory, antioxidant and liver function studies. All rats were obtained from the animal house colony of the National Organization for Drug Control and Research (NODCAR), Giza, Egypt. They were housed under standardized conditions of temperature and humidity and kept under a standard laboratory diet, which consisted of vitamin (1%), mineral mixture (4%), corn oil (10%), sucrose (20%), cellulose (0.2%), casein (95% pure) (10.5%) and starch (54.3%). Water was supplied ad libitum. Animal studies experiments were approved by the Animal Care and Use Committee of Faculty of Pharmacy, Cairo University following the World Medical Association Declaration of Helsinki [35]. Approval no: MP 1883 on October 2018.

#### 4.3.2. Determination of Median Lethal Dose (LD_50_)

LD_50_ of the ethanolic extract of the leaves of *A. thraskii* Baker applied the OECD-423 guidelines [29] in attempt to reduce the number of experimental animals used. Five Wistar albino rats of uniform weight were selected. After a full night of water fasting, one rat was given 2000 mg/kg b.wt. of the ethanolic test extract and kept under observation for 24 h for mortality. In case of the animal survival, the rest of the rats were tested sequentially. The close observation of the animals was continued for 14 days.

#### 4.3.3. In Vivo Antiarthritic Activity

A subplantar injection of 0.1 mL of complete Freund’s adjuvant (CFA) (Sigma chemical Co., St Louis, MO, USA) was used to induce arthritis into the right posterior paw under anesthesia (ketamine, 100 mg/kg, s.c). CFA consists of 10 mg/mL of heat-killed *Mycobacterium tuberculosis* in 0.15 mL mannide mono-oleate and 0.85 mL paraffin oil. Definite edema is produced within 24 h of injection. By the 9th day after inoculation, arthritis developed, and the next day, the treatments began and continued for 35 more days [36].

Seven groups of male albino rats were used (*n* = 6) as follows:

First group: served as a normal healthy control, receiving distilled water (3 mL/kg).

Second group: untreated arthritic rats served as negative control.

Third group: arthritic rats received MTX (as a reference anti-arthritic drug at a dose 2 mg/kg i.p. once weekly).

Fourth group: arthritic rats were injected by the plant extract (100 mg/kg/day b.wt. i.p. once daily) for 5 weeks.

Fifth group: arthritic rats were injected by the plant extract (200 mg/kg b.wt/day i.p. once daily) for 5 weeks.

Sixth group: arthritic rats were injected by the combined plant extract (100 mg/kg b.wt/day i.p. once daily) with MTX (2 mg/kg i.p. once weekly) for 5 weeks.

Seventh group: arthritic rats were injected by the combined plant extract (200 mg/kg b.wt/day i.p. once daily) with MTX (2 mg/kg i.p. once weekly) for 5 weeks.

#### 4.3.4. Biochemical Parameters

After 35 days of treatments, blood samples were drawn from the retro–orbital plexus under mild anesthesia with sodium pentobarbital. Sera were centrifuged for ten min and preserved at −20 °C for biomarker determination.

##### Diagnostic Rheumatoid Arthritis and Inflammation Markers

Enzyme-linked immunosorbent assay (ELISA) kits were used to measure the serum levels of TNF-α, NF-ĸB and IL-10 (Immuno-Biological Laboratories), rheumatoid factor RF (CUSBIO Biotech CO. Ltd., Hubei, China) and anti-CCP (EIAab Science Co. Ltd., Wuhan, China) following the manufactures’ protocol [37].

##### Determination of Oxidative Stress Mediators

The lipid peroxides malondialdehyde (MDA) was estimated colorimetrically by observing the reaction of the thiobarbituric acid-reacting substance (TBARS) with thiobarbituric acid (TBA) and measuring the absorbance of the resulting color at 532 nm. The total antioxidant capacity (TAC) was estimated by observing the reaction of the prepared phosphomolybdate dye with the sample, followed by a 90-min incubation at 95 °C and a 10-min cooling at room temperature, then measuring the absorbance at 695 nm wavelength. Meanwhile, the glutathione (GSH) was estimated by observing the reaction with Ellman’s reagent (5,5′-dithiobis (2-nitrobenzoic acid)) and measuring the resulting color at 412 nm. All of the previous colorimetric analysis adopted the methods described in the reports [38,39,40].

##### Determination of Markers for Liver Function Parameters

Collected serum samples were used as indices for liver function; serum alanine transaminase (ALT) and aspartate transaminase (AST) were measured by Reitman method. Alkaline phosphatase (ALP) was analyzed using disodium phenyl phosphate as a substrate [41]. Total bilirubin (TB) and total protein were estimated using a commercially available Accurex eco. Kit, following the producer’s information.

#### 4.3.5. Statistical Analysis

One-way of variance analysis (ANOVA) was used for statistical significance examination (mean ± SE, *n* = 6 animals) in Statistical Package for the Social Sciences (SPSS, version 22 software) followed by Duncan’s multiple range test (DMRT). A probability less than 0.05 was regarded as significant.

## 5. Conclusions

We concluded a synergetic combination of the leaf ethanolic extract of *A. thraskii* and MTX in the management of rheumatoid arthritis with fewer side effects on liver functions, as well as the possibility of the leaf extract to stand alone as an effective natural antiarthritic agent through antioxidant, anti-inflammatory and hepatoprotective mechanisms. The positive antirheumatic effect could be attributed to the isolated compounds. This finding supports and adds to the traditional use of *Aloe* as an anti-inflammatory remedy.

## Figures and Tables

**Table 1:** Effect of different treatments on the levels of diagnostic markers of rheumatoid arthritis (RF and anti-CCP).

Group	RF, Percent *	Anti-CCP, Percent *
Normal control	21.6 ± 0.67 ^a^	7.74 ± 0.36 ^a^
Negative control	78.4 ± 2.27 ^e^, 263.45% (↑)	43.1 ± 1.45 ^f^, 456.54% (↑)
MTX treated group	46.8 ± 2.31 ^c^, 40.3% (↓)	22.6 ± 0.75 ^c^, 47.6% (↓)
Ethanolic extract (100 mg/kg b.wt)	84.4 ± 2.22 ^e^, 7.7% (↓)	36.4 ± 2.91 ^e^, 15.6% (↓)
Ethanolic extract (200 mg/kg b.wt)	67.1 ± 3.11 ^d^, 14.4% (↓)	29.6 ± 3.05 ^d^, 31.4% (↓)
MTX + ethanolic extract (100 mg/kg b.wt)	41.5 ± 2.40 ^b^, 47% (↓)	20.4 ± 0.95 ^c^, 52.6% (↓)
MTX + ethanolic extract (200 mg/kg b.wt)	37.6 ± 2.15 ^bc^, 52% (↓)	15.3 ± 0.52 ^b^, 64.6% (↓)

RF, rheumatoid factor (mIU/mL); anti-CCP, anticyclic citrullinated peptide (IU/mL). * percentage increase or inhibition. Values are expressed as mean ± SE (*n* = 6 rats). In the same column, the presence of different letters (a–f) indicating a significant difference between groups using ANOVA in SPSS software (at *p* < 0.05), the letter “a” is the nearest result to the control while letter ”f” is the furthest from the control group.

**Table 2:** Effect of different treatments on the levels of markers of inflammation (NF-ĸB, TNF-α and IL-10).

Group	Proinflammatory Markers		Inflammatory Mediator
	NF-ĸB, Percent *	TNF-α, Percent *	IL_10_, Percent *
Normal control	4.56 ± 0.18 ^a^	36.9 ± 1.33 ^a^	98.6 ± 2.31 ^e^
Negative control	21.2 ± 0.65 ^e^, (364.38% ↑)	173.2 ± 3.23 ^e^, (370% ↑)	54.6 ± 1.85 ^a^, (44.69% ↓)
MTX treated group	8.65 ± 0.50 ^b^, (59.2% ↓)	93.4 ± 3.86 ^c^, (46.1%↑)	89.6 ± 2.09 ^d^, (64.2% ↑)
Ethanolic extract (100 mg/kg b.wt)	19.2 ± 1.09 ^d^, (9.2% ↓)	135.7 ± 3.81 ^d^, (21.6% ↓)	63.4 ± 2.46 ^b^, (16.3% ↑)
Ethanolic extract (200 mg/kg b.wt)	13.8 ± 0.59 ^c^, (35% ↓)	91.6 ± 3.83 ^c^, (47.1% ↓)	75.1 ± 3.31 ^c^, (37.7% ↑)
MTX + ethanolic extract (100 mg/kg b.wt)	8.29 ± 0.38 ^b^, (60.9% ↓)	83.5 ± 4.54 ^c^, (51.8% ↓)	95.8 ± 3.42 ^d,e^, (75.7%↑)
MTX + ethanolic extract (200 mg/kg b.wt)	7.66 ± 0.45 ^b^, (63.69% ↓)	69.4 ± 4.56 ^b^, (59.9% ↓)	99.3 ± 3.99 ^e^, (82% ↑)

NF-ĸB, nuclear factor kappa b (pg/mL); TNF-α, tumor necrosis factor (pg/mL); and IL-10, interleukin-10 (pg/mL). * Percentage increase or inhibition. Values are expressed as mean ± SE (*n* = 6 rats). In the same column, the presence of different letters (a–f) indicating a significant difference between groups using ANOVA in SPSS software (at *p* < 0.05), the letter “a” is the nearest result to the control while letter ”f” is the furthest from the control group.

**Table 3:** Effect of different treatments on levels of markers of oxidative stress (TAC, GSH and MDA).

Groups	TAC, Percent *	GSH, Percent *	MDA, Percent *
Normal control	0.459 ± 0.023 ^e^	49.7 ± 1.64 ^e^	13.4 ± 0.57 ^a^
Negative control	0.379 ± 0.018 ^c,d^, 17.3% (↓)	39.1 ± 1.68 ^c^, 21.4% (↓)	24.4 ± 0.92 ^c^, 22.7% (↑)
MTX treated group	0.251 ± 0.011 ^a^, 33.8% (↓)	24.3 ± 1.98 ^a^, 39.9% (↓)	32.6 ± 1.39 ^e^, 33.7% (↑)
Ethanolic extract (100 mg/kg b.wt)	0.416 ± 0.021 ^d,e^, 9.8% (↑)	43.5 ± 1.95 ^c,d^, 11.3% (↑)	19.6 ± 1.17 ^b^, 19.8% (↓)
Ethanolic extract (200 mg/kg b.wt)	0.436 ± 0.016 ^e^, 15% (↑)	47.3 ± 2.15 ^d,e^, 21.1% (↑)	14.8 ± 0.71 ^a^, 39.5% (↓)
MTX + ethanolic extract (100 mg/kg b.wt)	0.289 ± 0.011 ^a,b^, 23.8% (↓)	28.1 ± 1.26 ^a,b^, 28% (↓)	30.1 ± 1.31 ^d,e^, 23.2% (↑)
MTX + ethanolic extract (200 mg/Kg b.wt)	0.335 ± 0.012 ^b,c^, 11.8% (↓)	31.9 ± 1.87 ^b^, 18% (↓)	28.2 ± 1.80 ^d^, 15.5% (↑)

TAC, total antioxidant capacity (nmol/L); GSH, glutathione reduced (mg/dL); and MDA, malondialdehyde (nmol/mL). * percentage increase or inhibition. Values are expressed as mean ± SE (*n* = 6 rats). In the same column, the presence of different letters (a–f) indicating a significant difference between groups using ANOVA in SPSS software (at *p* < 0.05), the letter “a” is the nearest result to the control while letter ”f” is the furthest from the control group.

**Table 4:** Effect of different treatments on the markers of liver function (AST, ALT and ALP).

Group	AST, Percent *	ALT, Percent *	ALP, Percent *
Normal control	22.7 ± 1.28 ^a^	48.8 ± 1.35 ^a^	141.3 ± 6.75 ^a^
Negative control	50.8 ± 3.05 ^d^, 122% (↑)	116 ± 3.27 ^d^, 137.5% (↑)	232.9 ± 10.5 ^c^, 64.8% (↑)
MTX treated group	66.3 ± 2.52 ^f^_,_ 31.1% (↑)	159.8 ± 6.58 ^g^, 37.8% (↑)	378.3 ± 8.85 ^f^, 62.4% (↑)
Ethanolic extract (100 mg/Kg b.wt)	40.3 ± 1.33 ^c^, 19.9% (↓)	101.5 ± 3.22 ^c^,12.5% (↓)	176.8 ± 5.92 ^b^, 24.1% (↓)
Ethanolic extract (200 mg/Kg b.wt)	29.8 ± 1.17 ^b^, 40.7% (↓)	74.2 ± 2.41 ^b^, 36.1% (↓)	156.6 ± 3.98 ^a,b^, 32.8% (↓)
MTX + ethanolic extract (100 mg/Kg b.wt)	62.3 ± 2.96 ^e,f^, 23.8% (↑)	145.7 ± 3.80 ^f^, 25.6% (↑)	328.6 ± 7.33 ^e^, 41.1% (↑)
MTX + ethanolic extract (200 mg/Kg b.wt)	56.7 ± 1.94 ^e^, 12.6% (↑)	131 ± 5.7 ^e^, 12.6% (↑)	274.1 ± 13.2 ^d^, 17.1% (↑)

AST, aspartate amino transferase (U/mL); ALT, alanine transaminase (U/mL); and ALP, alkaline phosphatase (U/L).* percentage increase or inhibition. Values are expressed as mean ± SE (*n* = 6 rats). In the same column, the presence of different letters (a–f) indicating a significant difference between groups using ANOVA in SPSS software (at *p* < 0.05), the letter “a” is the nearest result to the control while letter ”f” is the furthest from the control group.

**Table 5:** Effect of different treatments on total bilirubin and total protein.

Groups	Total Bilirubin, Percent *	Total Protein, Percent *
Normal control	0.72 ± 0.03 ^a^	6.18 ± 0.25 ^d^
Negative control	1.31 ± 0.06 ^c^, 80.7% (↑)	4.78 ± 0.16 ^b,c^, 22.7% (↓)
MTX treated group	2.06 ± 0.07 ^f^, 58.9% (↑)	3.92 ± 0.11 ^a^, 18% (↓)
Ethanolic extract (100 mg/Kg b.wt)	1.05 ± 0.04 ^b^, 19% (↓)	5.08 ± 0.28 ^c^, 6.2% (↑)
Ethanolic extract (200 mg/Kg b.wt)	0.91 ± 0.037 ^b^, 31% (↓)	5.83 ± 0.22 ^d^, 22% (↑)
MTX + ethanolic extract (100 mg/Kg b.wt)	1.91 ± 0.06 ^e^, 43.1% (↑)	4.24 ± 0.18 ^b,c^, 11.3% (↓)
MTX + ethanolic extract (200 mg/Kg b.wt)	1.65 ± 0.07 ^d^, 27.1% (↑)	4.58 ± 0.16 ^a,b^, 4.2% (↓)

* percentage increase or inhibition. Total bilirubin (mg/dL); Total protein (g/dL) Values are expressed as mean ± SE (*n* = 6 rats). In the same column, the presence of different letters (a–f) indicating a significant difference between groups using ANOVA in SPSS software (at *p* < 0.05), the letter “a” is the nearest result to the control while letter ”f” is the furthest from the control group.

## Data Availability

No new data were created or analyzed in this study. Data sharing is not applicable to this article.

## Sample Availability

Samples of the compounds **1**–**5** are not available from the authors.
